# Advances of bispecific antibodies using/application in dermatology: a review

**DOI:** 10.3389/falgy.2025.1668931

**Published:** 2025-08-20

**Authors:** Ting Zhang, Yang Liu, Pei Xiong Liew, Yu Zhang, Zuotao Zhao

**Affiliations:** ^1^ Department of Dermatology, Tianjin Institute of Integrative Dermatology, Tianjin Academy of Traditional Chinese Medicine Affiliated Hospital, Tianjin, China; ^2^ Graduate School of Tianjin University of Traditional Chinese Medicine, Tianjin, China; ^3^ Immunology Center of Georgia, Department of Cellular Biology and Anatomy, Augusta University, Augusta, GA, United States

**Keywords:** bispecific antibodies, inflammatory skin diseases, skin tumors, skin infections, dermatology

## Abstract

Bispecific antibodies represent an important innovation in the field of biomedicine in recent years. Compared to monoclonal antibodies, their specific structure enables a single antibody molecule to bind to two different antigens simultaneously. This characteristic endows bispecific antibodies with more functions, regulating multiple signal pathways simultaneously, enhancing the therapeutic effect, and by infusion of targeted tumor antigens and drug carriers in advance, the contact time between the drug and normal tissues is reduced, and the toxic side effects are greatly reduced. They have shown promising application prospects, especially in dermatology and other fields. This article reviews the basic concepts of bispecific antibodies and their potential application in the treatment of skin diseases, including inflammatory skin diseases, skin tumors, and infectious skin diseases. The aim is to explore the current application status and future development directions of bispecific antibodies in dermatology, so as to provide references for related research and clinical practice.

## 1 Introductions

**Figure 1:**
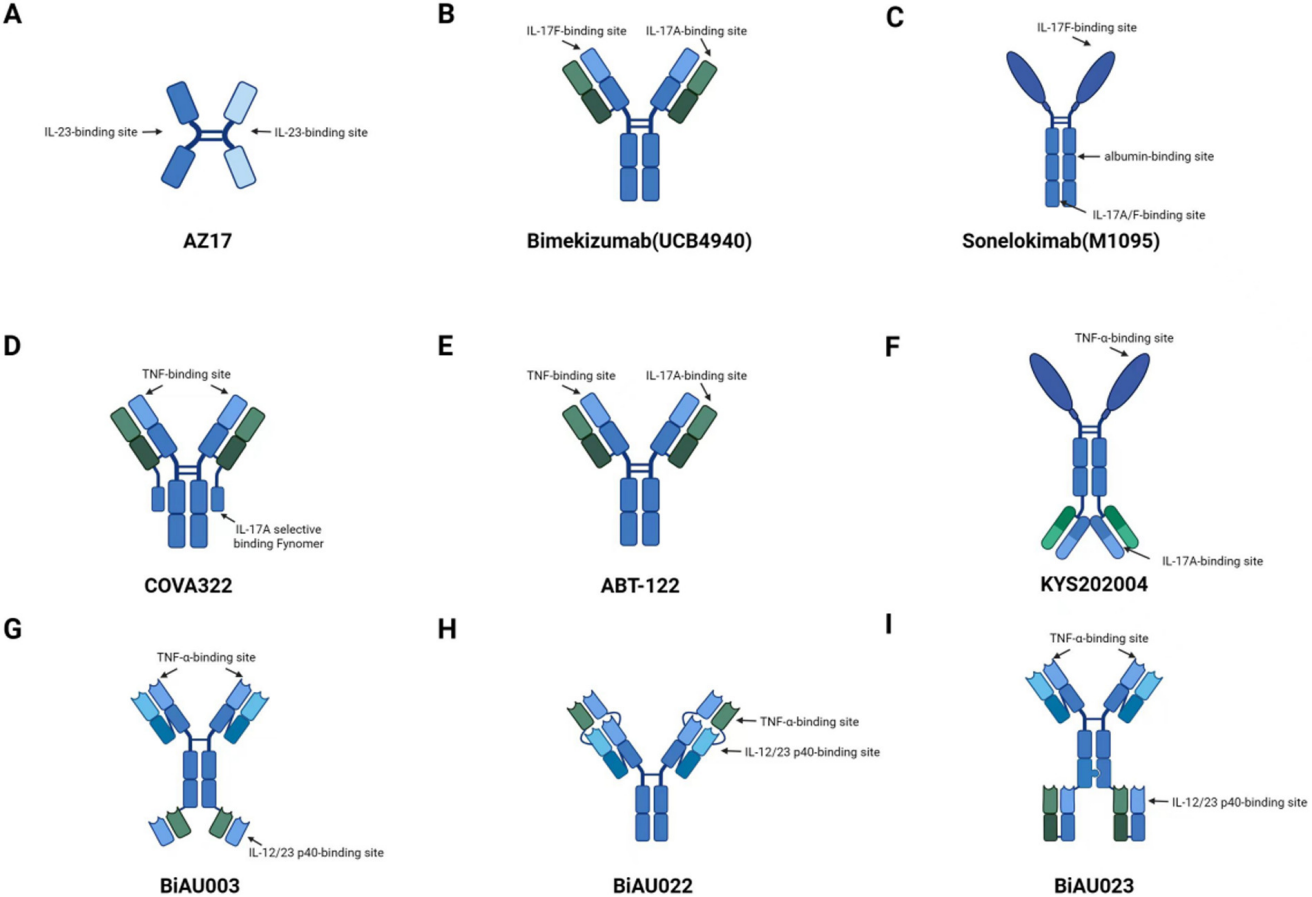
Current dual-specificity therapeutic drugs for psoriasis under development. **(A)** AZ17, composed of two single-chain variable fragments (scFvs), each binding to IL6 or IL23; **(B)** Bimekizumab, a humanized monoclonal antibody that simultaneously inhibits IL-17A and IL-17F; **(C)** Sonelokimab (M1095), a trivalent anti-IL-17A/F nanobody; **(D)** COVA322, composed of Fynomer selectively binding to IL-17A, fused with the C-terminal light chain of the anti-TNF-α antibody Adalimumab; **(E)** ABT-122, a dual-variable domain immunoglobulin targeting TNF-α and IL-17; **(F)** KYS202004, a dual-specificity fusion protein antagonizing TNF-α and IL-17A; **(G–I)** The connection methods of BiAU003, BiAU022, and BiAU023 are IgG-scFv, DVD-IgG, and IgG-Fab, respectively, all exhibiting high affinity for TNF-α and IL-12/23.

**Figure 2:**
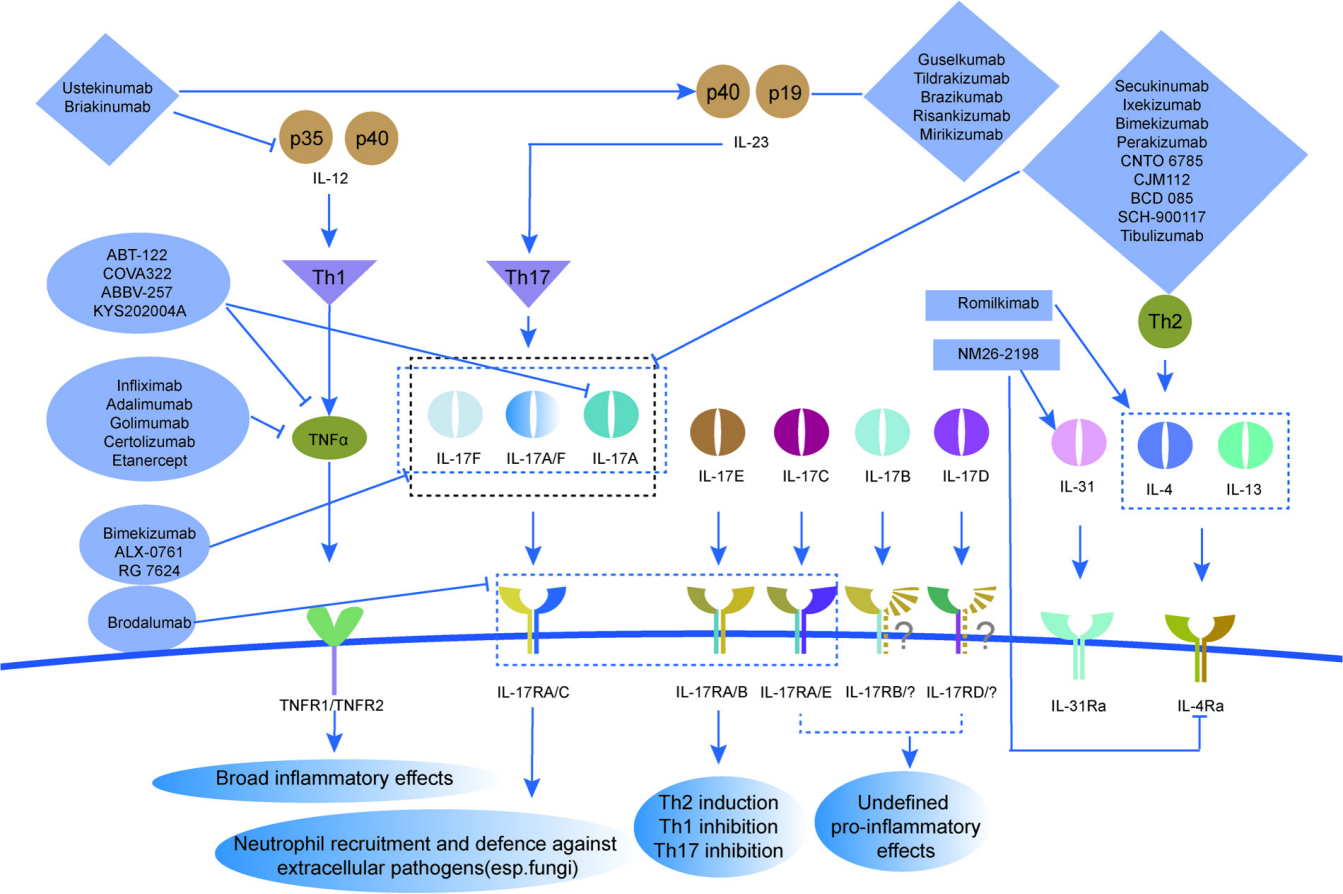
Immunoregulatory network of IL-12, IL-23, TNFα, and IL-17 family members in Th cell differentiation. These cytokines mediate effects like broad inflammatory responses, neutrophil recruitment against extracellular pathogens (e.g., fungi), and Th2 induction via receptors such as TNFR1/TNFR2 and IL-17RA/C. It also highlights therapeutic intervention points: Ustekinumab and Brakimumab target IL-12 (p35/p40), Guselkumab-class drugs target IL-23 (p40/p19), ABT-122 and COVA322 act on TNFα, and Bimekizumab targets IL-17A/F, showcasing the complexity of cytokine—mediated immune regulation and drug targeting.

Bispecific antibodies (BsAbs) are a class of antibodies that can simultaneously recognize and bind to two different antigens. In recent years, they have received extensive attention due to their potential applications in cancer immunotherapy and other diseases (1). The specific structure of BsAbs enables them to target two different molecules simultaneously irrespective if they are located on one cell or not, which enhances the therapeutic effect and potentially reducing side effects, for example, bispecific antibodies can be infused in advance with bispecific antibodies targeting tumor antigens and drug carriers using advance targeting tumor antigens and drug carriers, and then drug carriers can be infused. This method of administration can reduce the contact time between the drug and normal tissues, and greatly reduce the toxic side effects (2). The design of BsAbs generally falls into categories visualized in Figure 1 (structural formats, e.g., AZ17, Bimekizumab) and Figure 2 (pathway targeting, e.g., TNF-α/IL-17 axes). As shown, antibodies based on single—chain variable fragments (scFvs), like certain early—stage constructs not fully depicted here but following the scF vs. design principle, feature a compact structure with linked variable regions for dual—antigen recognition, enabling rapid tissue penetration. Full—length IgG—based antibodies, well—exemplified by Bimekizumab in Figure 1, retain the classic IgG architecture with two antigen—binding arms, allowing them to engage Fc receptors and trigger immune effector functions while binding to targets such as IL-17A/IL-17F. Fusion proteins, a category that can integrate diverse functional domains, might operate across pathways shown in Figure 2, like simultaneously interacting with cytokine—related targets in the TNF-α/IL-17 axes to modulate inflammatory cascades. These structural categories each bring distinct pharmacokinetic and pharmacodynamic properties, shaping their potential in dermatologic therapy. Among them, scFvs represent a minimal format of bispecific antibodies, typically composed of two distinct antigen-binding sites formed by linking the variable domains of immunoglobulin heavy (VH) and light (VL) chains via a flexible polypeptide linker. This format features a relatively small molecular weight and enhanced tissue penetration (2). However, due to the absence of an Fc region, scFvs generally have a shorter half-life. For example, the BiTE (bispecific T-cell engager) drug Blinatumomab (Blincyto, DrugBank® entry DB09052) has a mean (±SD) elimination half-life of only 1.25 ± 0.63 h, necessitating continuous intravenous (IV) infusion over a 4-week period to maintain sufficient therapeutic serum concentrations. In contrast, IgG-like bispecific antibodies retain the structural features of conventional IgG molecules, consisting of two antigen-binding fragments (Fabs) and one crystallizable fragment (Fc). Based on their design strategy, they can be categorized into symmetric and asymmetric structures. Asymmetric IgG-like bispecific antibodies contain two distinct Fv regions, such as Catumaxomab (anti-EpCAM × anti-CD3), which is produced using the quadroma technology. Symmetric IgG-like bispecific antibodies achieve bispecificity by attaching additional antigen-binding sites to the IgG molecule (3). The Fc region in IgG-like bispecific antibodies serves dual functions: it enables interaction with Fcγ receptors on immune cells to mediate immune responses such as antibody-dependent cellular cytotoxicity (ADCC), and it contributes to prolonged circulating half-life of the antibody *in vivo* (4).

The function and effect of bispecific antibodies is widely investigated in cancer therapy. BsAbs can close the gap between immune cells and tumor cells, induce the release of cytokines from immune cells, or target different signaling molecules to inhibit the growth and metastasis of tumors (5, 6). In recent years, candidate drugs have entered the clinical trial stage. Blincyto, a bispecific antibody targeting CD19 and CD3, has been approved for the treatment of acute lymphoblastic leukemia (7–11). Bispecific antibodies targeting different targets such as HER2 and CD3 are also being continuously developed (12, 13).

In the field of dermatology, however, monospecific antibodies remain the standard of care, particularly in the treatment of immune-mediated skin diseases such as psoriasis and atopic dermatitis. Approved therapies targeting single cytokines, including IL-17A, IL-23, and IL-4Rα, have demonstrated robust clinical efficacy and acceptable safety profiles. Nonetheless, bispecific antibodies are emerging as a promising next-generation strategy, especially in cases where single-target inhibition proves insufficient due to the redundancy and interplay of inflammatory pathways. BsAbs designed to simultaneously inhibit IL-17A and IL-17F, or IL-13 and TSLP, have entered clinical development and have shown favorable efficacy and safety signals. These agents are designed to achieve enhanced therapeutic outcomes and potentially reduce immunosuppressive side effects by offering a more comprehensive blockade of disease-driving cytokines.

Cytokines are key mediators produced by immune, epithelial, and endothelial cells that play central roles in the initiation and perpetuation of inflammatory skin conditions. In psoriasis, cytokines such as TNF-α, IL-17, and IL-23 are critical drivers of the IL-23/Th17 axis, which promotes keratinocyte hyperproliferation and inflammatory amplification through a feed-forward loop involving antimicrobial peptides, chemokines, and pro-inflammatory mediators (14–16). A summary of monoclonal antibodies and bispecific antibodies for the treatment of inflammatory skin diseases, including those targeting the above cytokines, is provided in Table 1. IL-23 promotes the differentiation and expansion of Th17 cells, thereby stimulating the secretion of pro-inflammatory cytokines such as IL-17A and IL-17F. IL-17 acts directly on keratinocytes, inducing the production of antimicrobial peptides (e.g., β-defensins), chemokines (e.g., CCL20), and other pro-inflammatory cytokines. This establishes a positive feedback loop of inflammation, which exacerbates skin lesion inflammation and contributes to the hyperproliferation of keratinocytes (17). As a classic pro-inflammatory cytokine, TNF-α not only enhances the activity of the IL-23/Th17 pathway but also activates the NF-κB signaling pathway, thereby further promoting the inflammatory cascade response (18, 19). Other cytokines such as IL-1β, IL-6, and IL-12 also play important roles in the inflammatory response of psoriasis, promoting the infiltration of immune cells and the release of inflammatory mediators (20). Bispecific antibodies achieve therapeutic purposes by targeting these cytokines (21–23).

**Table 1:** Summary of monoclonal antibodies and bispecific antibodies for the treatment of inflammatory skin diseases.

Antibody name	Type	Target	Indication	Developing company	Global highest R&D phase	Time of entering this phase
Secukinumab	Monoclonal Antibody	IL-17A	Psoriasis, Ankylosing Spondylitis, etc.	Novartis	Approved	January 2015
Ixekizumab	Monoclonal Antibody	IL-17A	Psoriasis, Psoriatic Arthritis, etc.	Eli Lilly	Approved	March 2016
Bimekizumab	Bispecific Antibody	IL-17A & IL-17F	Psoriasis	UCB	Approved	August 2021 (EU)
Brodalumab	Monoclonal Antibody	IL-17RA	Psoriasis	Amgen/AstraZeneca	Approved	February 2017
Ustekinumab	Monoclonal Antibody	IL-12/23p40	Psoriasis, Crohn's Disease, etc.	Janssen (J&J)	Approved	September 2009
Guselkumab	Monoclonal Antibody	IL-23p19	Psoriasis, Psoriatic Arthritis, etc.	Janssen (J&J)	Approved	July 2017
Tildrakizumab	Monoclonal Antibody	IL-23p19	Psoriasis	Sun Pharma	Approved	March 2018
Risankizumab	Monoclonal Antibody	IL-23p19	Psoriasis, Crohn's Disease, etc.	AbbVie	Approved	March 2019 (Japan)
Mirikizumab	Monoclonal Antibody	IL-23p19	Ulcerative Colitis, Crohn's Disease	Eli Lilly	Approved	April 2023 (FDA)
LY3090106/Tibulizumab	Bispecific Antibody	BAFF & IL-17A	Sjogren's Syndrome	Eli Lilly	Clinical Phase 2	December 2024
SAR156597/Romilkimab	Bispecific Antibody	IL-4 & IL-13	Systemic Sclerosis	Sanofi	Clinical Phase 2	January 2012
KYS202004A	Bispecific Antibody	IL-17A & TNF-α	Psoriasis, Rheumatoid Arthritis	Kyowa Kirin	Clinical Phase 1	December 2024
NM26-2198	Bispecific Antibody	IL-4Rα & IL-31	Atopic Dermatitis	Not Available	Clinical Phase 1	May 2023
ABT-122	Bispecific Antibody	IL-17A & TNF-α	Rheumatoid Arthritis	AbbVie	Development Terminated	–
Briakinumab	Monoclonal Antibody	IL-12/23p40	Psoriasis	Abbott	Development Terminated	–
Brazikumab	Monoclonal Antibody	IL-23p19	Crohn's Disease	Amgen/AstraZeneca	Preclinical Study	–

In the pathogenesis of atopic dermatitis, cytokines such as IL-4, IL-5, and IL-13 are secreted by Th2 cells which can promote the production and the infiltration of IgE, thus triggering the inflammatory response of the skin (24). IL-31, as a newly discovered cytokine, is closely related to the generation of itching sensation, further exacerbating the condition of patients (25). On the other hand, Th17 cytokines such as IL-17 and IL-22 also play important roles in the chronic stage of atopic dermatitis, promoting the damage of the skin barrier function and the persistence of inflammation (26). Bispecific antagonistic therapeutic strategies targeting these cytokines have shown good clinical effects (27–30). While current clinical use still relies heavily on monospecific antibodies, the development of BsAbs tailored to complex cytokine networks represents a forward-looking therapeutic strategy that may redefine the treatment paradigm for inflammatory skin diseases.

## 2 Treatment of skin diseases with traditional monospecific antibodies

Traditional monospecific antibody therapies—including monoclonal antibodies (mAbs) and polyclonal antibodies (pAbs)- have long been foundational in dermatologic treatment. Monoclonal antibodies, derived from a single B cell clone, exhibit high specificity and consistency, enabling targeted inhibition of disease-relevant molecules. In contrast, polyclonal antibodies are a heterogeneous mixture of immunoglobulins produced by multiple B cell clones which recognize multiple epitopes on a single or several antigens. Although pAbs lack the precision of mAbs, their broader reactivity can be advantageous in certain complex or polymicrobial disease contexts (32).

Clinically, mAbs have become central to the management of inflammatory skin disorders. Anti-TNF-α mAbs such as infliximab (31) and adalimumab (32, 33) are approved for moderate-to-severe psoriasis, effectively reducing inflammation by neutralizing TNF-α activity (15). Antibodies targeting IL-17 such as secukinumab (34), and those targeting IL-23, such as ustekinumab (35) have also shown excellent clinical efficacy (36). Ustekinumab has been shown to significantly relieve pruritus and erythema by modulating Th1 and Th17 immune responses (37). Other mAbs include rituximab, an anti-CD20 agent used in autoimmune dermatoses such as dermatomyositis and lupus, where B cell depletion reduces disease activity (38, 39). In cutaneous oncology, anti-PD-1 therapy such as pembrolizumab has improved melanoma outcomes by reactivating T cell responses (40). pAbs, though less widely used, remain relevant in specific contexts. Anti-IL-17 pAbs alleviate psoriatic symptoms via broad immunomodulation (41), and anti-IgE pAbs show promise in reducing allergic responses in atopic dermatitis (42, 43). Anti-venom and anti-infective pAbs assist in neutralizing toxins and pathogens in wounds and infections (44–47). However, both mAbs and pAbs act on single targets, which may be insufficient in complex diseases involving multiple inflammatory pathways. This limitation has led to growing interest in bispecific antibodies, which offer the ability to target two pathogenic mechanisms simultaneously.

## 3 Applications of bispecific antibodies in the field of oncology

BsAbs, which are capable of simultaneously recognizing two different antigens or epitopes, have demonstrated unique advantages and broad potential in the field of cancer therapy. Currently, the application of BsAbs in oncology can be broadly classified into several categories. The first category is T cell-redirecting BsAbs (e.g., CD3 × tumor antigen), which engage both T cells and tumor cells, promoting immune-mediated killing. Blinatumomab, targeting CD3 and CD19, has shown efficacy in acute lymphoblastic leukemia, significantly improving remission rates (7, 9, 10). Similarly, the HER2/CD3 bispecific antibody Zanidatamab effectively directs T cells against HER2-positive tumors and is approved for HER2-positive breast cancer (48, 49). In addition, a bispecific antibody targeting prostate-specific membrane antigen (PSMA) and CD3 has shown promising potential in the treatment of prostate cancer (50).

The second category includes dual-target inhibitory BsAbs that block two oncogenic pathways simultaneously, helping overcome resistance. ZEN003694-T, targeting EGFR and MET, is approved for EGFR exon 20-mutated NSCLC, offering a new strategy for refractory tumors. Tumor microenvironment-modulating BsAbs, like XmAb20717 (targeting PD-1 and CTLA-4), provide broader immune checkpoint inhibition than single-target mAbs, enhancing antitumor immunity (51, 52). It is under clinical trial for melanoma and other solid tumors.

Additionally, BsAbs can serve as targeted delivery vehicles, simultaneously binding tumor cells and chemotherapeutics to increase intratumoral drug accumulation while reducing off-target toxicity (53, 54).

## 4 Applications of bispecific antibodies in dermatology

The application of BsAbs in dermatology represents a rapidly advancing frontier in therapeutic innovation. Recent preclinical and clinical studies have demonstrated the potential of BsAbs in a range of IMIDs, including psoriasis, atopic dermatitis, systemic lupus erythematosus, systemic sclerosis, and primary Sjögren's syndrome. In these conditions, bsAbs have been designed to co-target key cytokines, immune cell receptors, or fibrotic mediators with aims to improve disease control, reduce treatment resistance, and minimize systemic immunosuppression. This approach holds particular promise in diseases where single-target therapies have shown suboptimal efficacy, making BsAbs as a strategic tool in the next generation of IMID treatment.

### 4.1 Psoriasis

Psoriasis is a chronic, relapsing, and inflammatory skin disease. In recent years, there has been an increasing number of studies on bispecific antibodies for psoriasis. The bispecific molecule AZ17 (Figure 1A) is generated by combining the high-affinity binding domains derived from monoclonal antibodies targeting human IL-6 and IL-23. AZ17 has been successfully tested in a mouse model. Compared with single anti-IL-6 or anti-IL-23 antibodies, it has shown greater efficacy in improving psoriatic-like inflammation and epidermal thickness (55).

Bimekizumab (UCB4940) (Figure 1B) is a novel humanized bispecific monoclonal IgG1 antibody. Adams et al. found that bimekizumab showed the same affinity for IL-17A as the commercially available ixekizumab and secukinumab, and demonstrated significant effects in the treatment of psoriasis (22). “In the study by Abdin et al., after treatment with bimekizumab, 76% of patients with moderate to severe plaque psoriasis achieved clearance within 72 h” (56). Concurrent with efficacy, mild injection site reactions (15.3%) and upper respiratory tract infections (10.2%) were observed, with no reports of severe infections or thromboembolic events (56). Sonelokimab (M1095) (Figure 1C) is a novel trivalent nanobody composed of monovalent camelid nanobodies specific for IL-17A, IL-17F, and human serum albumin. In a phase 2b study, 120 mg or lower doses of sonelokimab showed significant clinical benefits compared with the placebo. The incidence of adverse events in the 120 mg group was comparable to that in the placebo group (32.6% vs. 29.8%), with headache (6.8%) and nasopharyngitis (5.1%) being the most common; no severe allergic reactions or dose-limiting toxicities were reported (57). Silacci et al. constructed the bispecific TNF/IL-17A antibody COVA322 (Figure 1D), and it has completed phase I/II trials (NCT02243787) in subjects with stable moderate to severe chronic plaque psoriasis (58). *ABT-122* (Figure 1E) is a DVD-Ig bsAb that can also specifically neutralize human TNFα and IL-17A. Mease et al. conducted a phase II trial (NCT02349451) in subjects with active psoriatic arthritis who had an insufficient response to methotrexate. In this study, compared with the placebo, no serious infections or systemic hypersensitivity reactions were observed with ABT-122. Common adverse events included injection site erythema (8.7%) and diarrhea (4.3%), which were consistent with the safety profile of adalimumab (59). The efficacy of *ABT-122* was better than that of the placebo within 12 weeks, but there was no significant difference in efficacy between *ABT-122* and the anti-TNF*α* adalimumab (59, 60). In another long-term extension study for rheumatoid arthritis (RA) and psoriatic arthritis, the efficacy of *ABT-122* could be maintained for 36 weeks (61). KYS202004 (Figure 1F) is also a novel bispecific fusion protein that antagonizes TNF-α and IL-17A. In the psoriasis model, KYS202004A at a dose of 2 mg/kg had the same efficacy as the combination of ixekizumab and etanercept (62). In addition, Xu et al. designed bispecific antibodies BiAU003 (Figure 1G), BiAU022 (Figure 1H), and BiAU023 (Figure 1I) mainly based on the variable region sequences of adalimumab and ustekinumab antibodies. These antibodies can act as antagonists of TNF-α and IL-12/23 p40 and have a blocking effect on the formation of psoriasis in mice (63).

### 4.2 Atopic dermatitis

Atopic dermatitis (AD) is a complex chronic inflammatory skin disease, and its pathogenesis is closely related to the disorder of the immune system, especially the abnormal activation of T cells and the secretion of cytokines (64). Dupilumab is a monoclonal antibody targeting the IL-4 and IL-13 signaling pathways and has been widely used in the treatment of AD (65). In recent years, a number of clinical trials have confirmed the effectiveness and safety of Dupilumab in improving the skin symptoms and quality of life of patients with atopic dermatitis.

Dupilumab has shown excellent efficacy in reducing the area and severity of eczema and has relatively few side effects (66). In one clinical trial, Dupilumab showed significant efficacy. After patients received the treatment, the severity of skin lesions (EASI score) was significantly reduced, and the accompanying itching symptoms were also effectively relieved (67). Another study showed that after Dupilumab treatment, the quality of life assessment (DLQI score) of patients was significantly improved, and these effects remained stable during the duration of the treatment (68). In terms of safety, Dupilumab is well-tolerated, and the common adverse reactions are mainly injection site reactions and eye-related adverse events (69).

The long-term efficacy and safety of Dupilumab have also been further verified in an open-label extension study lasting 52 weeks (70). Patients can still maintain good efficacy after long-term use of Dupilumab (71). In addition, Tezepelumab is a novel bispecific antibody that can target thymic stromal lymphopoietin (TSLP) and IL-33 simultaneously and shows potential in the treatment of severe atopic dermatitis (72). IL-33 promotes the skin inflammatory response by activating Th2 cells and releasing pro-inflammatory cytokines. Simpson et al. found that Tezepelumab can significantly reduce the severity of eczema in patients and improve the overall condition of the skin. In their phase 2a trial, common adverse events were local erythema (8.2%) and headache (6.5%), with no treatment-related serious adverse events reported (73). Tezepelumab has also shown potential efficacy in patients with psoriasis, further demonstrating its broad application value in the treatment of skin diseases (74).

### 4.3 Systemic lupus erythematosus

Systemic lupus erythematosus (SLE) is a complex autoimmune disease, and its pathogenesis involves the abnormalities of multiple cytokines and immune responses. Firstly, IFN is considered an important pathological factor in SLE, especially type I IFN. Its level is significantly increased in SLE patients, promoting the activation of B cells and the production of antibodies (75). The abnormal activation of B cells and antibody production are one of the core characteristics of SLE. In addition, immunomodulatory factors such as TNF-α, IL-10, IL-17, and IL-6 also play important roles in the inflammatory response of SLE (76–78).

Obexelimab is a natural IgG bsAb that targets CD19 and FcγRIIb and inhibits the B-cell response (79). The co-ligation of CD19 and FcγRIIb inhibits key B-lineage cells in the pathogenesis of SLE. Merrill et al. conducted a double-blind, randomized, placebo-controlled phase II clinical trial. This study found that Obexelimab showed good efficacy in the treatment of SLE patients. The incidence of adverse events in the Obexelimab group was similar to that in the placebo group (65.0% vs. 62.3%), with fatigue (12.1%) and nasopharyngitis (10.3%) being the most common; no treatment-related serious adverse events occurred (80).

Telitacicept (RC18) is a novel BLyS/APRIL fusion protein, which is designed to target BAFF (BLyS) and APRIL simultaneously to regulate the survival and function of B cells. In animal models, RC18 has demonstrated good pharmacodynamic effects, which can significantly reduce the disease activity of SLE model mice, and the SLEDAI score decreases by more than 50% (81). Among the SLE patients treated with Telitacicept, 68% of the patients achieved the clinical improvement criteria (the SLEDAI score decreased by ≥4 points) within 12 weeks (82).

Zhang et al. first introduced the development of Rozibafusp alfa (AMG 570) in their study, pointing out that the drug aims to regulate the B cell-related immune response through the mechanism of action of simultaneously inhibiting inducible costimulatory ligand (ICOSL) and B cell activating factor (BAFF). This mechanism can effectively reduce the activation and proliferation of B cells in patients with autoimmune diseases, and then reduce the production of autoantibodies, which is a core pathological process of SLE. This study has laid the foundation for the application of AMG 570 in the treatment of SLE (83).

In the phase Ib/IIb, randomized, double-blind, placebo-controlled study conducted by Abuqayyas et al., Rozibafusp alfa did not significantly increase the incidence of adverse events during the treatment of active RA, and at the same time, it demonstrated its biological activity and therapeutic potential. This good safety and effectiveness support its further clinical application in the treatment of SLE (84, 85). Garces et al. suggested that innovative trials such as adaptive design and factor-rich design can increase the patient recruitment speed, reduce the discard rate, increase cost-effectiveness, and accelerate the marketing process of SLE drugs including Rozibafusp alfa (86). In addition, Blinatumoma is a bispecific anti-CD3/anti-CD19T-cell engager. Subklewe et al. first applied Blinatumomab to patients with rapidly progressive severe systemic sclerosis, and the patients' symptoms improved rapidly. However, the long-term treatment effect still needs further monitoring (87).

### 4.4 Systemic sclerosis

Systemic sclerosis (SSc) is a chronic autoimmune disease characterized by fibrosis, vasculopathy, and immune dysregulation (88). Recent advances have explored bispecific antibodies (bsAbs) as a novel therapeutic approach to modulate the complex immune landscape of SSc. By simultaneously targeting two antigens, BsAbs offer enhanced selectivity and synergistic immunomodulation.

Notably, bsAbs designed to simultaneously inhibit TGF-β signaling and IL-6 transduction-both implicated in fibrosis-have shown promise in preclinical studies, effectively attenuating fibroblast activation and extracellular matrix deposition (89, 90). Moreover, bsAbs targeting immune checkpoints and profibrotic cytokines are being investigated to reshape immune tolerance and reduce vascular inflammation. Another promising direction is the use of bsAbs to deplete dual-expressing pathogenic B cells or T cells while sparing regulatory subsets (91). While clinical data are still emerging, bsAb platforms such as DVD-Ig and CrossMab have laid the foundation for targeted multi-pathway blockade, representing a potential breakthrough in treating refractory SSc (92).

### 4.5 Sjögren's syndrome

Sjögren's syndrome (SS) is a systemic autoimmune disease primarily affecting exocrine glands, with B-cell hyperactivity and chronic inflammation as central features (93). The pathogenesis involves multiple immune pathways, including BAFF (B-cell activating factor), type I interferons, and co-stimulatory molecules (94). BsAbs have emerged as promising tools to intervene in this multifactorial network. Preclinical models have demonstrated that bsAbs targeting BAFF and ICOS-L or CD40l can effectively disrupt aberrant B-T cell interactions and reduce glandular infiltration (95). Additionally, BsAbs designed to simultaneously block IFNAR and TNFα signaling have been proposed to dampen inflammatory circuits more efficiently than monotherapies. Therapeutic formats such as bispecific T-cell engagers (BiTEs) or dual Fab BsAbs also allow for selective targeting of autoreactive immune cells (96). Although no BsAbs therapies for SS have yet reached phase 3 trials, early-phase studies indicate acceptable safety profiles and immunological efficacy, supporting further development. These approaches may redefine the therapeutic landscape of SS by offering tailored, combinatorial immunomodulation.

### 4.6 Skin tumors

In the treatment of skin tumors, especially melanoma, bispecific antibodies also show promising potential for clinical application. Bispecific antibodies that specifically target melanoma cells and T cells can effectively enhance the anti-tumor activity of T cells (97, 98). *M7824* is a bispecific antibody targeting PD-L1 and TGF-β, which has received extensive attention in the treatment of skin tumors such as melanoma in recent years. The combined treatment with M7824 can enhance the anti-tumor immune response, thereby improving the clinical efficacy. In Strauss et al.'s phase I trial, common adverse events included rash (22.4%) and diarrhea (18.1%), while grade ≥3 serious adverse events were mainly immune-related pneumonitis (3.2%) and elevated transaminases (2.8%) (99, 100).

Blinatumomab targets CD19 and CD3. Although it is mainly used for the treatment of acute lymphoblastic leukemia, its mechanism is also being studied for the treatment of melanoma. Studies have shown that Blinatumomab can effectively activate T cells and induce a cytotoxic response against melanoma cells (101). DuoBody-CD3 × CD20 is a novel bispecific antibody that can target CD20 and CD3 simultaneously. This antibody has shown significant anti-tumor activity in the skin B-cell lymphoma model, being able to effectively activate T cells and eliminate tumor cells (102, 103).

REGN1979 is also a bispecific antibody targeting CD20 and CD3. Studies on patients with cutaneous lymphoma have shown that this drug can effectively induce the apoptosis of tumor cells and achieve complete remission in some patients (104, 105). For a comprehensive overview of bispecific antibodies in dermatological research, including their targets, indications, and development stages, see Table 2. Although bispecific antibodies show good prospects in the treatment of skin tumors, further research is still needed to optimize their efficacy and safety. How to overcome the immunosuppressive factors in the tumor microenvironment is also an important issue for improving the efficacy of bispecific antibodies.

**Table 2:** Summary of bispecific antibodies in dermatological research.

Antibody name	Targets	Indication	Development stage	Company	Key safety data	References
Bimekizumab	IL-17A/IL-17F	Psoriasis	Approved (EU)	UCB	Injection site reactions (15.3%), upper respiratory infections (10.2%); no severe infections or thromboembolic events	(56)
Tezepelumab	TSLP/IL-33	Atopic Dermatitis	Phase 2	Amgen	Local erythema (8.2%), headache (6.5%); no treatment-related serious adverse events	(73)
Obexelimab	CD19/Fc*γ*RIIb	Systemic Lupus Erythematosus	Phase 2	Xencor	Fatigue (12.1%), nasopharyngitis (10.3%); no treatment-related SAEs	(80)
M7824	PD-L1/TGF-*β*	Melanoma	Phase 2	Merck KGaA	Rash (22.4%), diarrhea (18.1%); grade ≥3 SAEs: immune-related pneumonitis (3.2%), elevated transaminases (2.8%)	(100)
DuoBody-CD3xCD20	CD20/CD3	Cutaneous B-cell Lymphoma	Phase 1	Genmab	Cytokine release syndrome (5.7%), fever (4.2%)	(102)
REGN1979	CD20/CD3	Cutaneous Lymphoma	Phase 1	Regeneron	Infusion-related reactions (7.3%), neutropenia (2.1%)	(104)
Anti-S.aureus bispecific HCAb	LukS-PV/LukF-PV	S. aureus Skin Infections	Preclinical	Undisclosed	No significant toxicity in murine models; no immunosuppression observed	(106)

### 4.7 Skin infections

Bispecific antibodies against specific pathogens can simultaneously target infected cells and immune cells, thereby improving the efficiency of immune clearance. *Staphylococcus aureus (S. aureus)* is one of the main pathogens causing skin infections. Bispecific antibodies against *S. aureus* can effectively neutralize its toxins and activate the host immune system at the same time. Laventie et al. designed an engineered tetravalent bispecific HCAb against *S. aureus* PVL in immunotransgenic mice, which neutralizes toxin activity by simultaneously binding to both LukS-PV and LukF-PV subunits, thereby preventing their oligomerization and pore formation on host immune cells, especially neutrophils (106). Moreover, a study by Tkaczyk et al. compared the methods of multi-mechanism monoclonal antibodies (Mabs) against *S. aureus α*-toxin and clumping factor A (ClfA) with engineered bispecific antibodies and found that the combination of Mabs targeting ClfA and *α*-toxin was more promising than the corresponding BiSAb (107).

*Candida spp.* infections are particularly common in immunosuppressed patients. Bispecific antibodies against *Candida* can simultaneously target fungal cell wall components and immune cells, enhancing the antifungal immune response. Zito et al. constructed a bispecific antibody MP65/bglu mAb against fungal microorganisms, which can simultaneously recognize β-glucan and MP65 determinants in *Candida* and can be used as a biomarker for *Candida* (108). *Herpes simplex virus (HSV)* often causes skin and mucosal infections. Bispecific antibodies against *HSV* can simultaneously recognize viral surface glycoproteins and host immune cells, enhancing the ability to clear the virus. Ravirala et al. found that the combined use of bispecific and trispecific antibodies with *HSV*-based oncolytic virus therapy can improve the anti-tumor effect by enhancing T cell recruitment and activation through CD3 and tumor-associated antigens, while also engaging co-stimulatory receptors such as 4-1BB, thereby amplifying the cytotoxic immune response and improving tumor clearance (109). Renard et al. also confirmed the ability of the bispecific antibody CD3/EGFR bimAb to redirect T cells against *HPV in vitro* transformed keratinocytes in an autologous three-dimensional culture model.

## 5 Bsabs advantages of bispecific antibodies

Bispecific antibodies have demonstrated increasing potential in the treatment of skin diseases, and their advantages are mainly reflected in aspects such as targeted therapy, enhanced immune response, and improved bioavailability of drugs. Firstly, they are capable of simultaneously targeting two different cytokines or antigens, thereby regulating the immune response more effectively. Moreover, by targeting multiple pathological mechanisms, bispecific antibodies can reduce the potential side effects that may occur in single-target therapy. For example, in patients with psoriasis, more thorough inhibition of inflammation can be achieved by simultaneously targeting TNF-α and IL-17, which is difficult to accomplish in traditional antibody therapy (110). Secondly, bispecific antibodies can promote the apoptosis of target cells by forming immune complexes and enhance the clearance function of immune cells, and this has been proven effective in cancer treatment (5, 111). Bispecific antibodies can also enhance the body's anti-tumor immune response by regulating the immune microenvironment (112). In recent years, the application of bispecific antibodies in cancer immunotherapy has also provided new ideas for the treatment of skin diseases.

In addition, the design of bispecific antibodies can optimize their pharmacokinetic properties, prolong the half-life, and improve the bioavailability (4, 113, 114). With the development of genetic engineering technology, the production processes and purification techniques of BsAbss have been continuously optimized, making their applications in drug development increasingly widespread (115, 116). Overall, the multi-targeting characteristics and flexibility of bispecific antibodies make them a promising therapeutic option, especially when facing complex tumor heterogeneity and immune escape mechanisms.

## 6 Prospects and limitations

In the field of dermatology, bispecific antibodies, as an innovative biological treatment method, have demonstrated broad application prospects and significant clinical value. With the rapid development of biotechnology, bispecific antibodies not only provide new tools in basic research, promoting a deeper understanding of the mechanisms of various skin diseases, but also show good therapeutic effects in clinical applications, especially in refractory skin diseases, inflammatory skin diseases, and skin tumors.

However, the research, development, and application of bispecific antibodies still face many challenges, such as high production costs, issues related to drug stability and tolerability (5, 115, 117–119). Therefore, future research should focus on optimizing antibody design and production processes to improve their economic efficiency and clinical adaptability. At the same time, conducting large-scale clinical trials to further evaluate the long-term effects and safety of bispecific antibodies in dermatological applications is also an important task in the future.

## References

[1] Hollander N. Bispecific antibodies for cancer therapy. Immunotherapy. (2009) 1(2):211–22. 10.2217/1750743X.1.2.211 20635943

[2] Chen Y Shu X Zhao Y Zhang B Ma Z Zhang H. Single chain antibody fragment display systems: a review. Sheng Wu Gong Cheng Xue Bao. (2023) 39(9):3681–94. 10.13345/j.cjb.220911 37805846

[3] Portell CA Wenzell CM Advani AS. Clinical and pharmacologic aspects of blinatumomab in the treatment of B-cell acute lymphoblastic leukemia. Clin Pharmacol. (2013) 5(Suppl 1):5–11. 23671399 10.2147/CPAA.S42689 PMC3650887

[4] Brinkmann U Kontermann RE. The making of bispecific antibodies. MAbs. (2017) 9(2):182–212. 10.1080/19420862.2016.1268307 28071970 PMC5297537

[5] Wei J Yang Y Wang G Liu M. Current landscape and future directions of bispecific antibodies in cancer immunotherapy. Front Immunol. (2022) 13:1035276. 10.3389/fimmu.2022.1035276 36389699 PMC9650279

[6] Demaria O Cornen S Daëron M Morel Y Medzhitov R Vivier E. Harnessing innate immunity in cancer therapy. Nature. (2019) 574(7776):45–56. 10.1038/s41586-019-1593-5 31578484

[7] Brown PA Ji L Xu X Devidas M Hogan LE Borowitz MJ Effect of postreinduction therapy consolidation with blinatumomab vs chemotherapy on disease-free survival in children, adolescents, and young adults with first relapse of B-cell acute lymphoblastic leukemia: a randomized clinical trial. JAMA. (2021) 325(9):833–42. 10.1001/jama.2021.0669 33651090 PMC7926290

[8] Foà R Bassan R Vitale A Elia L Piciocchi A Puzzolo MC Dasatinib–blinatumomab for Ph-positive acute lymphoblastic leukemia in adults. N Engl J Med. (2020) 383(17):1613–23. 10.1056/NEJMoa2016272 33085860

[9] Gökbuget N Dombret H Bonifacio M Reichle A Graux C Faul C Blinatumomab for minimal residual disease in adults with B-cell precursor acute lymphoblastic leukemia. Blood. (2018) 131(14):1522–31. 10.1182/blood-2017-08-798322 29358182 PMC6027091

[10] Kantarjian H Stein A Gökbuget N Fielding AK Schuh AC Ribera JM Blinatumomab versus chemotherapy for advanced acute lymphoblastic leukemia. N Engl J Med. (2017) 376(9):836–47. 10.1056/NEJMoa1609783 28249141 PMC5881572

[11] Locatelli F Zugmaier G Rizzari C Morris JD Gruhn B Klingebiel T Effect of blinatumomab vs chemotherapy on event-free survival among children with high-risk first-relapse B-cell acute lymphoblastic leukemia: a randomized clinical trial. JAMA. (2021) 325(9):843–54. 10.1001/jama.2021.0987 33651091 PMC7926287

[12] Long AW Xu H Santich BH Guo H Hoseini SS de Stanchina E Heterodimerization of T cell engaging bispecific antibodies to enhance specificity against pancreatic ductal adenocarcinoma. J Hematol Oncol. (2024) 17(1):20. 10.1186/s13045-024-01538-5 38650005 PMC11036555

[13] Oberg HH Peipp M Kellner C Sebens S Krause S Petrick D Novel bispecific antibodies increase γδ T-cell cytotoxicity against pancreatic cancer cells. Cancer Res. (2014) 74(5):1349–60. 10.1158/0008-5472.CAN-13-0675 24448235

[14] Blauvelt A Chiricozzi A. The immunologic role of IL-17 in psoriasis and psoriatic arthritis pathogenesis. Clin Rev Allergy Immunol. (2018) 55(3):379–90. 10.1007/s12016-018-8702-3 30109481 PMC6244934

[15] Sieminska I Pieniawska M Grzywa TM. The immunology of psoriasis–current concepts in pathogenesis. Clin Rev Allergy Immunol. (2024) 66(2):164–91. 10.1007/s12016-024-08991-7 38642273 PMC11193704

[16] Singh JA Guyatt G Ogdie A Gladman DD Deal C Deodhar A Special article: 2018 American college of rheumatology/national psoriasis foundation guideline for the treatment of psoriatic arthritis. Arthritis Rheumatol. (2019) 71(1):5–32. 10.1002/art.40726 30499246 PMC8218333

[17] Liu T Li S Ying S Tang S Ding Y Li Y The IL-23/IL-17 pathway in inflammatory skin diseases: from bench to bedside. Front Immunol. (2020) 11:594735. 10.3389/fimmu.2020.594735 33281823 PMC7705238

[18] Lee Y Choi HK N'Deh KPU Choi YJ Fan M Kim EK Inhibitory effect of Centella asiatica extract on DNCB-induced atopic dermatitis in HaCaT cells and BALB/c mice. Nutrients. (2020) 12(2):411. 10.3390/nu12020411 32033291 PMC7071208

[19] Yamaguchi HL Yamaguchi Y Peeva E. Role of innate immunity in allergic contact dermatitis: an update. Int J Mol Sci. (2023) 24(16):12975. 10.3390/ijms241612975 37629154 PMC10455292

[20] Shahi A Afzali S Amirzargar A Mohaghegh P Salehi S Mansoori Y. Potential roles of inflammasomes in the pathophysiology of psoriasis: a comprehensive review. Mol Immunol. (2023) 161:44–60. 10.1016/j.molimm.2023.06.007 37481828

[21] Tokuyama M Mabuchi T. New treatment addressing the pathogenesis of psoriasis. Int J Mol Sci. (2020) 21(20):7488. 10.3390/ijms21207488 33050592 PMC7589905

[22] Adams R Maroof A Baker T Lawson ADG Oliver R Paveley R Bimekizumab, a novel humanized IgG1 antibody that neutralizes both IL-17A and IL-17F. Front Immunol. (2020) 11:1894. 10.3389/fimmu.2020.01894 32973785 PMC7473305

[23] Liu Z Liu H Xu P Yin Q Wang Y Opoku YK Ameliorative effects of a fusion protein dual targeting interleukin 17A and tumor necrosis factor α on imiquimod-induced psoriasis in mice. Biomed Pharmacother. (2018) 108:1425–34. 10.1016/j.biopha.2018.09.178 30372845

[24] Dubin C Del Duca E Guttman-Yassky E. The IL-4, IL-13 and IL-31 pathways in atopic dermatitis. Expert Rev Clin Immunol. (2021) 17(8):835–52. 10.1080/1744666X.2021.1940962 34106037

[25] Orfali RL Aoki V. Blockage of the IL-31 pathway as a potential target therapy for atopic dermatitis. Pharmaceutics. (2023) 15(2):577. 10.3390/pharmaceutics15020577 36839897 PMC9961325

[26] Sugaya M. The role of Th17-related cytokines in atopic dermatitis. Int J Mol Sci. (2020) 21(4):1314. 10.3390/ijms21041314 32075269 PMC7072946

[27] Maggi L Mazzoni A Capone M Liotta F Annunziato F Cosmi L. The dual function of ILC2: from host protection to pathogenic players in type 2 asthma. Mol Aspects Med. (2021) 80:100981. 10.1016/j.mam.2021.100981 34193344

[28] Olaguibel JM Sastre J Rodríguez JM Del Pozo V. Eosinophilia induced by blocking the IL-4/IL-13 pathway: potential mechanisms and clinical outcomes. J Investig Allergy Clin Immunol. (2022) 32(3):165–80. 10.18176/jiaci.0823 35522053

[29] Di Lernia V. Therapeutic strategies in extrinsic atopic dermatitis: focus on inhibition of IL-4 as a new pharmacological approach. Expert Opin Ther Targets. (2015) 19(1):87–96. 10.1517/14728222.2014.965682 25283256

[30] Huang R Hu L Jiang F. Study of cytokine-induced immunity in bullous pemphigoid: recent developments. Ann Med. (2023) 55(2):2280991. 10.1080/07853890.2023.2280991 38109924 PMC10732206

[31] Langley RG Elewski BE Lebwohl M Reich K Griffiths CE Papp K Secukinumab in plaque psoriasis—results of two phase 3 trials. N Engl J Med. (2014) 371(4):326–38. 10.1056/NEJMoa1314258 25007392

[32] Blauvelt A Papp KA Griffiths CE Randazzo B Wasfi Y Shen YK Efficacy and safety of guselkumab, an anti-interleukin-23 monoclonal antibody, compared with Adalimumab for the continuous treatment of patients with moderate to severe psoriasis: results from the phase III, double-blinded, placebo- and active comparator-controlled VOYAGE 1 trial. J Am Acad Dermatol. (2017) 76(3):405–17. 10.1016/j.jaad.2016.11.041 28057360

[33] Jang M Koh I Lee JE Lim JY Cheong JH Kim P. Increased extracellular matrix density disrupts E-cadherin/β-catenin complex in gastric cancer cells. Biomater Sci. (2018) 6(10):2704–13. 10.1039/C8BM00843D 30151505

[34] Yokota N Kondo M Hayashi A Ichishi M Matsushima Y Nakanishi T Psoriasis-like skin rash triggered by a local infection in a patient with eosinophilic granulomatosis with polyangiitis that was well controlled by mepolizumab treatment. Clin Case Rep. (2023) 11(6):e7532. 10.1002/ccr3.7532 37305885 PMC10256866

[35] Huszti Z. Relative efficacy and safety of ustekinumab compared to anti-TNF-alfa therapies in patients with active psoriatic arthritis. Value Health. (2014) 17(7):A373. 10.1016/j.jval.2014.08.2575 27200804

[36] Schinocca C Rizzo C Fasano S Grasso G La Barbera L Ciccia F Role of the IL-23/IL-17 pathway in rheumatic diseases: an overview. Front Immunol. (2021) 12:637829. 10.3389/fimmu.2021.637829 33692806 PMC7937623

[37] Pan Y Xu L Qiao J Fang H. A systematic review of ustekinumab in the treatment of atopic dermatitis. J Dermatolog Treat. (2018) 29(6):539–41. 10.1080/09546634.2017.1406894 29164954

[38] Kolesnik M Becker E Reinhold D Ambach A Heim MU Gollnick H Treatment of severe autoimmune blistering skin diseases with combination of protein A immunoadsorption and rituximab: a protocol without initial high dose or pulse steroid medication. J Eur Acad Dermatol Venereol. (2014) 28(6):771–80. 10.1111/jdv.12175 23651052

[39] Zhen C Hou Y Zhao B Ma X Dai T Yan C. Efficacy and safety of rituximab treatment in patients with idiopathic inflammatory myopathies: a systematic review and meta-analysis. Front Immunol. (2022) 13:1051609. 10.3389/fimmu.2022.1051609 36578492 PMC9791086

[40] Robert C Ribas A Schachter J Arance A Grob JJ Mortier L Pembrolizumab versus ipilimumab in advanced melanoma (KEYNOTE-006): *post-hoc* 5-year results from an open-label, multicentre, randomised, controlled, phase 3 study. Lancet Oncol. (2019) 20(9):1239–51. 10.1016/S1470-2045(19)30388-2 31345627

[41] Lipman NS Jackson LR Trudel LJ Weis-Garcia F. Monoclonal versus polyclonal antibodies: distinguishing characteristics, applications, and information resources. ILAR J. (2005) 46(3):258–68. 10.1093/ilar.46.3.258 15953833

[42] Sánchez-Rodríguez G Puig L. Pathogenic role of IL-17 and therapeutic targeting of IL-17F in psoriatic arthritis and spondyloarthropathies. Int J Mol Sci. (2023) 24(12):1035. 10.3390/ijms241210305 37373452 PMC10299014

[43] Ghoreschi K Balato A Enerbäck C Sabat R. Therapeutics targeting the IL-23 and IL-17 pathway in psoriasis. Lancet. (2021) 397(10275):754–66. 10.1016/S0140-6736(21)00184-7 33515492

[44] Antivenom slithers back to life. Nat Biotechnol. (2024) 42(4):537–8. 10.1038/s41587-024-02221-3 38570693

[45] Alangode A Rajan K Nair BG. Snake antivenom: challenges and alternate approaches. Biochem Pharmacol. (2020) 181:114135. 10.1016/j.bcp.2020.114135 32628928

[46] Ke S Kil H Roggy C Shields T Quinn Z Quinn AP Potential therapeutic targets for combination antibody therapy against *Staphylococcus aureus* infections. Antibiotics. (2024) 13(11):1046. 10.3390/antibiotics13111046 39596740 PMC11591076

[47] Moragues MD Rementeria A Sevilla MJ Eraso E Quindos G. *Candida* antigens and immune responses: implications for a vaccine. Expert Rev Vaccines. (2014) 13(8):1001–12. 10.1586/14760584.2014.932253 24957934

[48] Huang S van Duijnhoven SMJ Sijts A van Elsas A. Bispecific antibodies targeting dual tumor-associated antigens in cancer therapy. J Cancer Res Clin Oncol. (2020) 146(12):3111–22. 10.1007/s00432-020-03404-6 32989604 PMC7679314

[49] Xiang X Wang J Lu D Xu X. Targeting tumor-associated macrophages to synergize tumor immunotherapy. Signal Transduct Target Ther. (2021) 6(1):75. 10.1038/s41392-021-00484-9 33619259 PMC7900181

[50] Dorff T Horvath LG Autio K Bernard-Tessier A Rettig MB Machiels JP A phase I study of acapatamab, a half-life extended, PSMA-targeting bispecific T-cell engager for metastatic castration-resistant prostate cancer. Clin Cancer Res. (2024) 30(8):1488–500. 10.1158/1078-0432.CCR-23-2978 38300720 PMC11395298

[51] Gao X Xu N Li Z Shen L Ji K Zheng Z Safety and antitumour activity of cadonilimab, an anti-PD-1/CTLA-4 bispecific antibody, for patients with advanced solid tumours (COMPASSION-03): a multicentre, open-label, phase 1b/2 trial. Lancet Oncol. (2023) 24(10):1134–46. 10.1016/S1470-2045(23)00411-4 37797632

[52] Zhao Y Ma Y Fan Y Zhou J Yang N Yu Q A multicenter, open-label phase Ib/II study of cadonilimab (anti PD-1 and CTLA-4 bispecific antibody) monotherapy in previously treated advanced non-small-cell lung cancer (AK104-202 study). Lung Cancer. (2023) 184:107355. 10.1016/j.lungcan.2023.107355 37677918

[53] Brivio E Bautista F Zwaan CM. Naked antibodies and antibody-drug conjugates: targeted therapy for childhood acute lymphoblastic leukemia. Haematologica. (2024) 109(6):1700–12. 10.3324/haematol.2023.283815 38832425 PMC11141655

[54] Beishenaliev A Loke YL Goh SJ Geo HN Mugila M Misran M Bispecific antibodies for targeted delivery of anti-cancer therapeutic agents: a review. J Control Release. (2023) 359:268–86. 10.1016/j.jconrel.2023.05.032 37244297

[55] Stenderup K Rosada C Shanebeck K Brady W Van Brunt MP King G AZ17: a new bispecific drug targeting IL-6 and IL-23 with potential clinical use—improves psoriasis in a human xenograft transplantation model. Protein Eng Des Sel. (2015) 28(10):467–80. 10.1093/protein/gzv034 26271488

[56] Abdin R Gharib R Bunick CG Issa NT. Rapid remission of plaque psoriasis with bimekizumab treatment. J Drugs Dermatol. (2024) 23(8):694–6. 10.36849/JDD.8381 39093648

[57] Papp KA Weinberg MA Morris A Reich K. IL17A/F Nanobody sonelokimab in patients with plaque psoriasis: a multicentre, randomised, placebo-controlled, phase 2b study. Lancet. (2021) 397(10284):1564–75. 10.1016/S0140-6736(21)00440-2 33894834

[58] Silacci M Lembke W Woods R Attinger-Toller I Baenziger-Tobler N Batey S Discovery and characterization of COVA322, a clinical-stage bispecific TNF/IL-17A inhibitor for the treatment of inflammatory diseases. MAbs. (2016) 8(1):141–9. 10.1080/19420862.2015.1093266 26390837 PMC4966518

[59] Mease PJ Genovese MC Weinblatt ME Peloso PM Chen K Othman AA Phase II study of ABT-122, a tumor necrosis factor- and interleukin-17A-targeted dual Variable domain immunoglobulin, in patients with psoriatic arthritis with an inadequate response to methotrexate. Arthritis Rheumatol. (2018) 70(11):1778–89. 10.1002/art.40579 29855175 PMC6221045

[60] Khatri A Klünder B Peloso PM Othman AA. Exposure-response analyses demonstrate no evidence of interleukin 17A contribution to efficacy of ABT-122 in rheumatoid or psoriatic arthritis. Rheumatology. (2019) 58(2):352–60. 10.1093/rheumatology/key312 30376130

[61] Genovese MC Weinblatt ME Mease PJ Aelion JA Peloso PM Chen K Dual inhibition of tumour necrosis factor and interleukin-17A with ABT-122: open-label long-term extension studies in rheumatoid arthritis or psoriatic arthritis. Rheumatology. (2018) 57(11):1972–81. 10.1093/rheumatology/key173 30032191

[62] Liu Z Song L Yang J Liu H Zhang Y Pi X Discovery and preclinical evaluation of KYS202004A, a novel bispecific fusion protein targeting TNF-α and IL-17A, in autoimmune disease models. Int Immunopharmacol. (2024) 136:112383. 10.1016/j.intimp.2024.112383 38843642

[63] Xu P Xie N Ye C. Inhibitory effect of bispecific antibody targeting IL-12 p40 and TNF-α simultaneously on psoriasis in mice. Xi Bao Yu Fen Zi Mian Yi Xue Za Zhi. (2017) 33(7):890–5. 28712395

[64] Sroka-Tomaszewska J Trzeciak M. Molecular mechanisms of atopic dermatitis pathogenesis. Int J Mol Sci. (2021) 22(8):4130. 10.3390/ijms22084130 33923629 PMC8074061

[65] Bitton A Avlas S Reichman H Itan M Karo-Atar D Azouz NP A key role for IL-13 signaling via the type 2 IL-4 receptor in experimental atopic dermatitis. Sci Immunol. (2020) 5(44):eaaw2938. 10.1126/sciimmunol.aaw2938 32060143

[66] Blauvelt A Teixeira HD Simpson EL Costanzo A De Bruin-Weller M Barbarot S Efficacy and safety of upadacitinib vs. dupilumab in adults with moderate-to-severe atopic dermatitis: a randomized clinical trial. JAMA Dermatol. (2021) 157(9):1047–55. 10.1001/jamadermatol.2021.3023 34347860 PMC8340015

[67] Blauvelt A de Bruin-Weller M Gooderham M Cather JC Weisman J Pariser D Long-term management of moderate-to-severe atopic dermatitis with dupilumab and concomitant topical corticosteroids (LIBERTY AD CHRONOS): a 1-year, randomised, double-blinded, placebo-controlled, phase 3 trial. Lancet. (2017) 389(10086):2287–303. 10.1016/S0140-6736(17)31191-1 28478972

[68] Reich K Thyssen JP Blauvelt A Eyerich K Soong W Rice ZP Efficacy and safety of abrocitinib versus dupilumab in adults with moderate-to-severe atopic dermatitis: a randomised, double-blind, multicentre phase 3 trial. Lancet. (2022) 400(10348):273–82. 10.1016/S0140-6736(22)01199-0 35871814

[69] Silverberg JI Thyssen JP Fahrbach K Mickle K Cappelleri JC Romero W Comparative efficacy and safety of systemic therapies used in moderate-to-severe atopic dermatitis: a systematic literature review and network meta-analysis. J Eur Acad Dermatol Venereol. (2021) 35(9):1797–810. 10.1111/jdv.17351 33991374 PMC8453983

[70] Merola JF Sidbury R Wollenberg A Chen Z Zhang A Shumel B Dupilumab prevents flares in adults with moderate to severe atopic dermatitis in a 52-week randomized controlled phase 3 trial. J Am Acad Dermatol. (2021) 84(2):495–7. 10.1016/j.jaad.2020.05.003 32387659

[71] Gooderham MJ Hong HC Eshtiaghi P Papp KA. Dupilumab: a review of its use in the treatment of atopic dermatitis. J Am Acad Dermatol. (2018) 78(3 Suppl 1):S28–36. 10.1016/j.jaad.2017.12.022 29471919

[72] Borgia F Custurone P Li Pomi F Vaccaro M Alessandrello C Gangemi S. IL-33 and IL-37: a possible axis in skin and allergic diseases. Int J Mol Sci. (2023) 24(1):372. 10.3390/ijms24010372 PMC9820694 36613827

[73] Simpson EL Parnes JR She D Crouch S Rees W Mo M Tezepelumab, an anti-thymic stromal lymphopoietin monoclonal antibody, in the treatment of moderate to severe atopic dermatitis: a randomized phase 2a clinical trial. J Am Acad Dermatol. (2019) 80(4):1013–21. 10.1016/j.jaad.2018.11.059 30550828

[74] Shi L Yu M Jin Y Chen P Mu G Tam SH A novel monoclonal antibody against human thymic stromal lymphopoietin for the treatment of TSLP-mediated diseases. Front Immunol. (2024) 15:1442588. 10.3389/fimmu.2024.1442588 39726595 PMC11670205

[75] Hamilton JA Hsu HC Mountz JD. Role of production of type I interferons by B cells in the mechanisms and pathogenesis of systemic lupus erythematosus. Discov Med. (2018) 25(135):21–9. 29466691

[76] Jin S Yu C Yu B. Changes of serum IL-6, IL-10 and TNF-α levels in patients with systemic lupus erythematosus and their clinical value. Am J Transl Res. (2021) 13(4):2867–74. 34017450 PMC8129414

[77] Biswas S Bieber K Manz RA. IL-10 revisited in systemic lupus erythematosus. Front Immunol. (2022) 13:970906. 10.3389/fimmu.2022.970906 35979356 PMC9376366

[78] Koga T Ichinose K Kawakami A Tsokos GC. The role of IL-17 in systemic lupus erythematosus and its potential as a therapeutic target. Expert Rev Clin Immunol. (2019) 15(6):629–37. 10.1080/1744666X.2019.1593141 30874446

[79] Szili D Cserhalmi M Bankó Z Nagy G Szymkowski DE Sármay G. Suppression of innate and adaptive B cell activation pathways by antibody coengagement of Fc*γ*RIIb and CD19. MAbs. (2014) 6(4):991–9. 10.4161/mabs.28841 24828435 PMC4171032

[80] Merrill JT Guthridge J Smith M June J Koumpouras F Machua W Obexelimab in systemic lupus erythematosus with exploration of response based on gene pathway co-expression patterns: a double-blind, randomized, placebo-controlled, phase 2 trial. Arthritis Rheumatol. (2023) 75(12):2185–94. 10.1002/art.42652 37459248

[81] Yao X Ren Y Zhao Q Chen X Jiang J Liu D Pharmacokinetics analysis based on target-mediated drug distribution for RC18, a novel BLyS/APRIL fusion protein to treat systemic lupus erythematosus and rheumatoid arthritis. Eur J Pharm Sci. (2021) 159:105704. 10.1016/j.ejps.2021.105704 33440243

[82] Fan Y Gao D Zhang Z. Telitacicept, a novel humanized, recombinant TACI-Fc fusion protein, for the treatment of systemic lupus erythematosus. Drugs Today. (2022) 58(1):23–32. 10.1358/dot.2022.58.1.3352743 35107091

[83] Zhang M Lee F Knize A Jacobsen F Yu S Ishida K Development of an ICOSL and BAFF bispecific inhibitor AMG 570 for systemic lupus erythematosus treatment. Clin Exp Rheumatol. (2019) 37(6):906–14. 30789152

[84] Abuqayyas L Chen PW Dos Santos MT Parnes JR Doshi S Dutta S Pharmacokinetics and pharmacokinetic/pharmacodynamic properties of rozibafusp alfa, a bispecific inhibitor of BAFF and ICOSL: analyses of phase I clinical trials. Clin Pharmacol Ther. (2023) 114(2):371–80. 10.1002/cpt.2929 37150935

[85] Abuqayyas L Cheng LE Teixeira Dos Santos M Sullivan BA Ruiz-Santiago N Wang H Safety and biological activity of rozibafusp alfa, a bispecific inhibitor of inducible costimulator ligand and B cell activating factor, in patients with rheumatoid arthritis: results of a phase 1b, randomized, double-blind, placebo-controlled, multiple ascending dose study. ACR Open Rheumatol. (2022) 4(10):903–11. 10.1002/acr2.11487 35899378 PMC9555197

[86] Garces S Karis E Merrill JT Askanase AD Kalunian K Mo M Improving resource utilisation in SLE drug development through innovative trial design. Lupus Sci Med. (2023) 10(2):e000890. 10.1136/lupus-2022-000890 37491104 PMC10373732

[87] Subklewe M Magno G Gebhardt C Bücklein V Szelinski F Arévalo HJR Application of blinatumomab, a bispecific anti-CD3/CD19T-cell engager, in treating severe systemic sclerosis: a case study. Eur J Cancer. (2024) 204:114071. 10.1016/j.ejca.2024.114071 38691878

[88] Denton CP Khanna D. Systemic sclerosis. Lancet. (2017) 390(10103):1685–99. 10.1016/S0140-6736(17)30933-9 28413064

[89] Lafyatis R. Transforming growth factor β—at the centre of systemic sclerosis. Nat Rev Rheumatol. (2014) 10(12):706–19. 10.1038/nrrheum.2014.137 25136781

[90] Bohdziewicz A Pawlik KK Maciejewska M Sikora M Alda-Malicka R Czuwara J Future treatment options in systemic sclerosis—potential targets and ongoing clinical trials. J Clin Med. (2022) 11(5):1310. 10.3390/jcm11051310 35268401 PMC8911443

[91] Beesley CF Goldman NR Taher TE Denton CP Abraham DJ Mageed RA Dysregulated B cell function and disease pathogenesis in systemic sclerosis. Front Immunol. (2023) 13:999008. 10.3389/fimmu.2022.999008 36726987 PMC9885156

[92] Jiang A Liu N Wang J Zheng X Ren M Zhang W The role of PD-1/PD-L1 axis in idiopathic pulmonary fibrosis: friend or foe? Front Immunol. (2022) 13:1022228. 10.3389/fimmu.2022.1022228 36544757 PMC9760949

[93] Brito-Zerón P Retamozo S Ramos-Casals M. Síndrome de sjögren. Med Clin. (2023) 160(4):163–71. 10.1016/j.medcli.2022.10.007 36528400

[94] Nocturne G Mariette X. B cells in the pathogenesis of primary Sjögren syndrome. Nat Rev Rheumatol. (2018) 14(3):133–45. 10.1038/nrrheum.2018.1 29416129

[95] Samy E Wax S Huard B Hess H Schneider P. Targeting BAFF and APRIL in systemic lupus erythematosus and other antibody-associated diseases. Int Rev Immunol. (2017) 36(1):3–19. 10.1080/08830185.2016.1276903 28215100

[96] Wang Z Wang G Lu H Li H Tang M Tong A. Development of therapeutic antibodies for the treatment of diseases. Mol Biomed. (2022) 3(1):35. 10.1186/s43556-022-00100-4 36418786 PMC9684400

[97] Märkl F Benmebarek MR Keyl J Cadilha BL Geiger M Karches C Bispecific antibodies redirect synthetic agonistic receptor modified T cells against melanoma. J Immunother Cancer. (2023) 11(5):e006436. 10.1136/jitc-2022-006436 37208128 PMC10201273

[98] Tang J Gong Y Ma X. Bispecific antibodies progression in malignant melanoma. Front Pharmacol. (2022) 13:837889. 10.3389/fphar.2022.837889 35401191 PMC8984188

[99] Brauchle E Kasper J Daum R Schierbaum N Falch C Kirschniak A Biomechanical and biomolecular characterization of extracellular matrix structures in human colon carcinomas. Matrix Biol. (2018) 68–69:180–93. 10.1016/j.matbio.2018.03.016 29605717

[100] Strauss J Heery CR Schlom J Madan RA Cao L Kang Z Phase I trial of M7824 (MSB0011359C), a bifunctional fusion protein targeting PD-L1 and TGFβ, in advanced solid tumors. Clin Cancer Res. (2018) 24(6):1287–95. 10.1158/1078-0432.CCR-17-2653 29298798 PMC7985967

[101] Baeuerle PA Wesche H. T-cell-engaging antibodies for the treatment of solid tumors: challenges and opportunities. Curr Opin Oncol. (2022) 34(5):552–8. 10.1097/CCO.0000000000000869 35880455 PMC9415207

[102] Engelberts PJ Hiemstra IH de Jong B Schuurhuis DH Meesters J Beltran Hernandez I DuoBody-CD3xCD20 induces potent T-cell-mediated killing of malignant B cells in preclinical models and provides opportunities for subcutaneous dosing. EBioMedicine. (2020) 52:102625. 10.1016/j.ebiom.2019.102625 31981978 PMC6992935

[103] Brachelente C Affolter VK Fondati A Porcellato I Sforna M Lepri E CD3 and CD20 coexpression in a case of canine cutaneous epitheliotropic T-cell lymphoma (mycosis fungoides). Vet Pathol. (2016) 53(3):563–6. 10.1177/0300985815604724 26354309

[104] Smith EJ Olson K Haber LJ Varghese B Duramad P Tustian AD A novel, native-format bispecific antibody triggering T-cell killing of B-cells is robustly active in mouse tumor models and cynomolgus monkeys. Sci Rep. (2015) 5:17943. 10.1038/srep17943 26659273 PMC4675964

[105] Tun AM Ansell SM. Immunotherapy in Hodgkin and non-Hodgkin lymphoma: innate, adaptive and targeted immunological strategies. Cancer Treat Rev. (2020) 88:102042. 10.1016/j.ctrv.2020.102042 32521386

[106] Laventie BJ Rademaker HJ Saleh M de Boer E Janssens R Bourcier T Heavy chain-only antibodies and tetravalent bispecific antibody neutralizing *Staphylococcus aureus* leukotoxins. Proc Natl Acad Sci U S A. (2011) 108(39):16404–9. 10.1073/pnas.1102265108 21930905 PMC3182740

[107] Tkaczyk C Kasturirangan S Minola A Jones-Nelson O Gunter V Shi YY Multimechanistic monoclonal antibodies (MAbs) targeting *Staphylococcus aureus* alpha-toxin and clumping factor A: activity and efficacy comparisons of a MAb combination and an engineered bispecific antibody approach. Antimicrob Agents Chemother. (2017) 61(8):e00629-17. 10.1128/AAC.00629-17 28584141 PMC5527613

[108] Zito A Bromuro C Mandili G Chiani P Horenstein AL Malavasi F A murine, bispecific monoclonal antibody simultaneously recognizing β-glucan and MP65 determinants in Candida Species. PLoS One. (2016) 11(2):e0148714. 10.1371/journal.pone.0148714 26859561 PMC4747543

[109] Ravirala D Mistretta B Gunaratne PH Pei G Zhao Z Zhang X. Co-delivery of novel bispecific and trispecific engagers by an amplicon vector augments the therapeutic effect of an HSV-based oncolytic virotherapy. J Immunother Cancer. (2021) 9(7):e002454. 10.1136/jitc-2021-002454 34230110 PMC8261877

[110] Hawkes JE Chan TC Krueger JG. Psoriasis pathogenesis and the development of novel targeted immune therapies. J Allergy Clin Immunol. (2017) 140(3):645–53. 10.1016/j.jaci.2017.07.004 28887948 PMC5600287

[111] Trabolsi A Arumov A Schatz JH. Bispecific antibodies and CAR-T cells: dueling immunotherapies for large B-cell lymphomas. Blood Cancer J. (2024) 14(1):27. 10.1038/s41408-024-00997-w 38331870 PMC10853226

[112] Klein C Brinkmann U Reichert JM Kontermann RE. The present and future of bispecific antibodies for cancer therapy. Nat Rev Drug Discov. (2024) 23(4):301–19. 10.1038/s41573-024-00896-6 38448606

[113] Stadler CR Ellinghaus U Fischer L Bähr-Mahmud H Rao M Lindemann C Preclinical efficacy and pharmacokinetics of an RNA-encoded T cell-engaging bispecific antibody targeting human claudin 6. Sci Transl Med. (2024) 16(748):eadl2720. 10.1126/scitranslmed.adl2720 38776391

[114] Fu C Tong W Yu L Miao Y Wei Q Yu Z When will the immune-stimulating antibody conjugates (ISACs) be transferred from bench to bedside? Pharmacol Res. (2024) 203:107160. 10.1016/j.phrs.2024.107160 38547937

[115] Goebeler ME Stuhler G Bargou R. Bispecific and multispecific antibodies in oncology: opportunities and challenges. Nat Rev Clin Oncol. (2024) 21(7):539–60. 10.1038/s41571-024-00905-y 38822215

[116] Spiess C Zhai Q Carter PJ. Alternative molecular formats and therapeutic applications for bispecific antibodies. Mol Immunol. (2015) 67(2 Pt A):95–106. 10.1016/j.molimm.2015.01.003 25637431

[117] Shim H. Bispecific antibodies and antibody-drug conjugates for cancer therapy: technological considerations. Biomolecules. (2020) 10(3):360. 10.3390/biom10030360 32111076 PMC7175114

[118] Suurs FV Lub-de Hooge MN de Vries EGE de Groot DJA. A review of bispecific antibodies and antibody constructs in oncology and clinical challenges. Pharmacol Ther. (2019) 201:103–19. 10.1016/j.pharmthera.2019.04.006 31028837

[119] Wang Q Chen Y Park J Liu X Hu Y Wang T Design and production of bispecific antibodies. Antibodies. (2019) 8(3):43. 10.3390/antib8030043 31544849 PMC6783844

